# Management of a Septic Open Abdomen Patient with Spontaneous Jejunal Perforation after Emergent C/S with Confounding Factor of Mild Acute Pancreatitis

**DOI:** 10.1155/2016/7153579

**Published:** 2016-02-23

**Authors:** Fahri Yetisir, Akgün Ebru Sarer, Hasan Zafer Acar, Gokhan Osmanoglu, Mehmet Özer, Faik Yaylak

**Affiliations:** ^1^General Surgery Department, Atatürk Research and Training Hospital, Turkey; ^2^Anesthesiology and Reanimation Department, Atatürk Research and Training Hospital, Turkey; ^3^Natomed Private Hospital General Surgery Department, Turkey; ^4^General Surgery Department, Medical Park Private Hospital, Turkey

## Abstract

*Introduction*. We report the management of a septic Open Abdomen (OA) patient by the help of negative pressure therapy (NPT) and abdominal reapproximation anchor (ABRA) system in pregnant woman with spontaneous jejunal perforation after emergent cesarean section (C/S) with confounding factor of mild acute pancreatitis (AP).* Presentation of Case*. A 29-year-old and 34-week pregnant woman with AP underwent C/S. She was arrested after anesthesia induction and responded to cardiopulmonary resuscitation (CPR). There were only ash-colored serosanguinous fluid within abdomen during C/S. After C/S, she was transferred to intensive care unit (ICU) with vasopressor support. On postoperative 1st day, she underwent reoperation due to fecal fluid coming near the drainage. Leakage point could not be identified exactly and operation had to be deliberately abbreviated due to hemodynamic instability. NPT was applied. Two days later source control was provided by conversion of enteroatmospheric fistula (EAF) to jejunostomy. ABRA was added and OA was closed. No hernia developed at 10-month follow-up period.* Conclusion*. NPT application in septic OA patient may gain time to patient until adequate source control could be achieved. Using ABRA in conjunction with NPT increases the fascial closure rate in infected OA patient.

## 1. Introduction

Acute pancreatitis (AP) is a rare event in pregnancy (3/10 000) including a wide range of situations ranging from mild pancreatitis to serious one. Rate of AP is correlated directly with advancing gestational age. Older reviews of AP in pregnancy reported maternal and fetal mortality rates as high as 20 and 50%, respectively [1]. Contrary to this, Hernandez et al. reported 34 cases of AP with no maternal deaths and a fetal loss of only 4.7% [2].

The incidence of bowel injuries is 0.08% in cesarean section (C/S). Patients with a history of abdominal surgery scar are at high risk for intestinal injuries [3]. After C/S there was very rare spontaneous cecal perforation but there was no spontaneous small bowel perforation in the literature [4].

Enterocutaneous fistulas (ECF) are one of the most devastating abdominal complications in intra-abdominal surgery. A newly defined complication is called enteroatmospheric fistula (EAF) which is an enteric fistula in the middle of an Open Abdomen (OA) [5]. The OA is defined in World Society of Abdominal Compartment Syndrome guideline as one that requires a temporary abdominal closure due to the skin and fascia not being closed after laparotomy [6]. OA management is a life-saving and challenging strategy in situations such as the abdominal compartment syndrome (ACS) and damage-control surgery in severe generalized peritonitis [7, 8]. Mortality rates up to 50% were reported and even higher in the infected OA [9, 10].

We would like to report the management of a septic OA patient by the help of dynamic abdominal closure systems in a 34-week pregnant woman with spontaneous jejunal perforation developing after emergent C/S with her confounding factor of mild acute pancreatitis.

## 2. Presentation of Case

**Figure 1:**
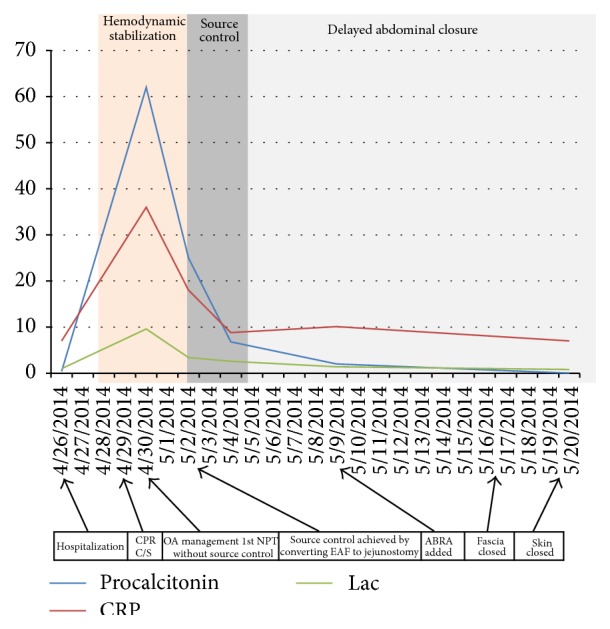
All the important interventions are given on the scale and change in the CRP, lactate, and procalcitonin level according to these interventions from hospitalization to closure of OA.

**Figure 2:**
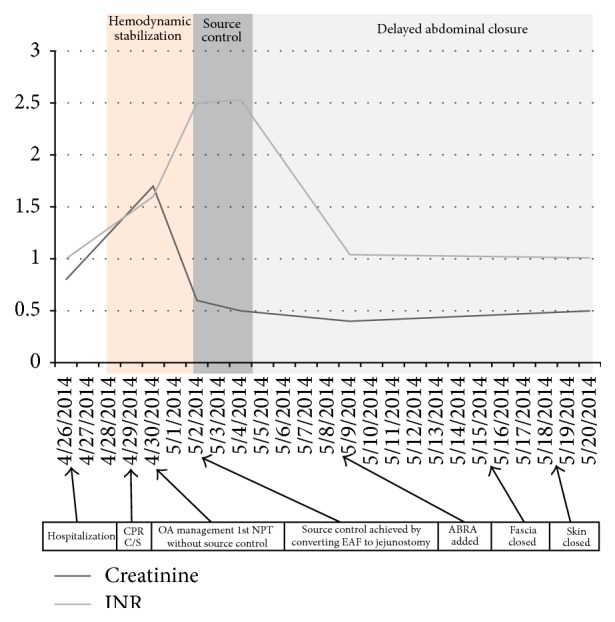
All the important interventions are given on the scale and change in the creatinine and INR level according to these interventions from hospitalization to closure of OA.

**Figure 3:**
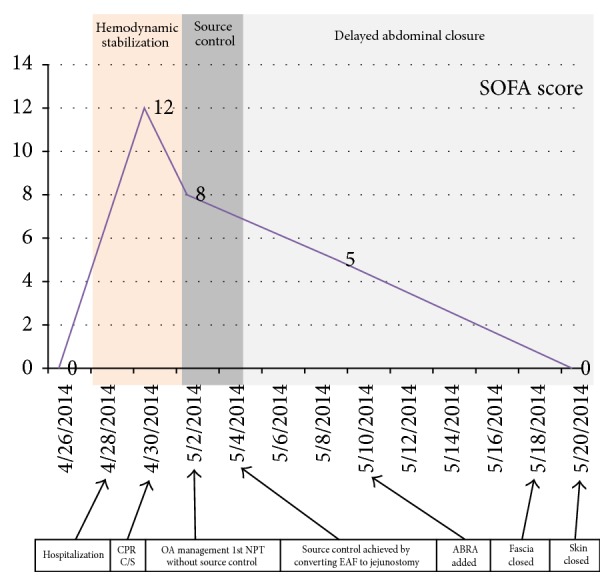
All the important interventions are given on the scale and change in the SOFA score according to these interventions from hospitalization to closure of OA.

A 29-year-old and 34-week pregnant woman was admitted to emergency department with the complaint of abdominal pain, nausea, and vomiting for three days. Epigastric pain radiating to back was present. In her past history, she had been operated on for perforated appendicitis and C/S 10 and 5 years ago, respectively. Her vital parameters were as fallows: blood pressure (BP): 100/50 mmHg, heart rate (HR): 94, and fever: 36.7°C. On her abdominal examination, badly healed midline incision scar from xiphoid to pubis and C/S scar were present. Peritoneal signs and rebound tenderness were positive at epigastric region. In biochemical analysis, LDH: 277 U/L, lipase: 256 U/L, Amylase: 288 U/L, AST: 81 U/L, ALT: 101 U/L, CRP: 7 mg/dL, and in total blood count WBC: 12,000 K/*μ*L. Multiple small calculi in different sizes were seen and pancreas was evaluated as edematous suboptimally in abdominal US. The patient was admitted to service to observe and treat for mild AP. The patient underwent C/S emergently for fetal distress 3 days after hospitalization. The patient had a cardiac arrest just after anesthesia induction. She responded to cardiopulmonary resuscitation (CPR) performed approximately for 15 minutes. Fetus was dead on delivery and did not respond to neonatal resuscitation. After C/S, on abdominal exploration, it seemed that there was ash-colored serosanguinous fluid and excessive visceral adhesions. After C/S, the patient was transferred to ICU with vasopressor support. The patient was consulted to us for fecal fluid at the drainage site on postoperative 1st day. At that time her vital parameters under high dose of vasopressor treatment were as follows: BP: 80/45 mmHg, HR: 154 F: 37.9, and RR: 34; her laboratory values were as follows: LDH: 729 U/L, creatinine: 1.6 mg/dL, Alb: 1.61 g/dL, WBC: 2.400 K/uL, CRP: 36.8 mg/dL (0–0.8), procalcitonin: 62.1 ng/mL (<0.5), INR: 1.4, and D-dimer > 1000 ng/mL. In arterial blood gas analysis, pH was 7.24, pCO_2_ was 34, pO_2_ was 159, HCO_3_ was 14, BE was −12, and lac was 9.6. The patient was in septic-shock and metabolic acidosis. The increases in CRP, lactate, and procalcitonin values were shown in Figure 1; increases in creatinine and INR values were shown in Figure 2. SOFA score at that time was 12 (Figure 3) and expected mortality was 45% accordingly.

**Figure 4:**
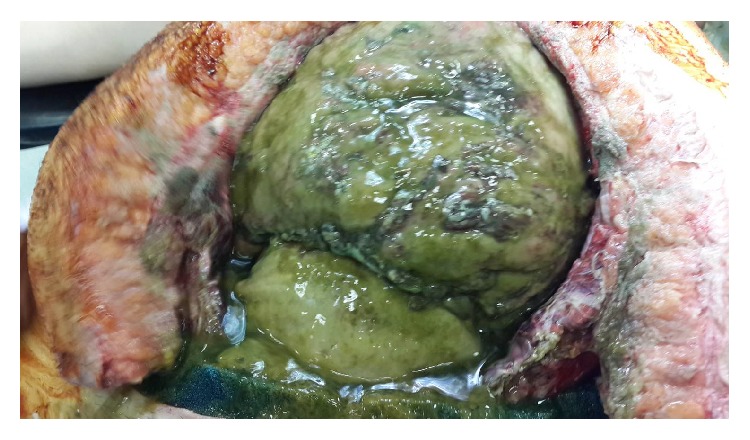
After midline incision was added, excessive amount of enteric influents, edematous and fragile intra-abdominal visceral tissue, and excessive adhesions are seen.

The patient underwent emergent operation from the same Pfannenstiel incision. Enteric influents were seen during exploration and midline incision was added to get better surgical exposure. Since intestines were edematous and fragile and excessive adhesions were present between intra-abdominal visceral tissues, anatomical structures were not identified exactly. During this time, enteric influents were coming from the nonvisible posterior part of adhesive intestinal tissue (Figure 4). Leakage point could not be identified exactly; operation had to be deliberately abbreviated due to hemodynamic instability and physiologic derangement of patient. After drainage was established, the abdomen was irrigated with saline and negative pressure therapy was applied.

**Figure 5:**
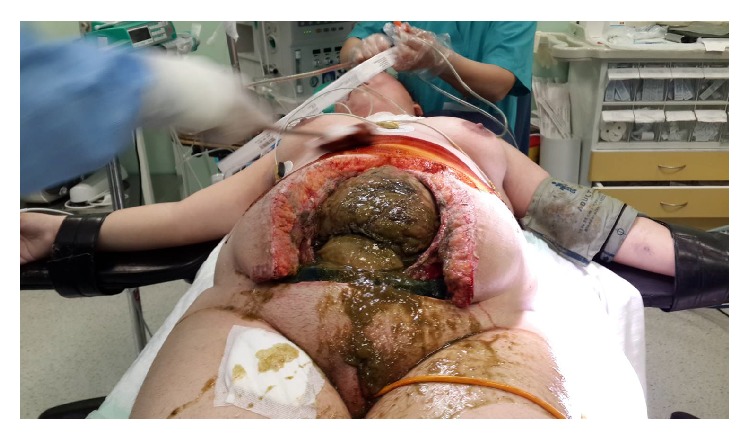
Excessive amount of enteric influents, edematous and fragile intra-abdominal visceral tissue, and excessive adhesions are seen.

**Figure 6:**
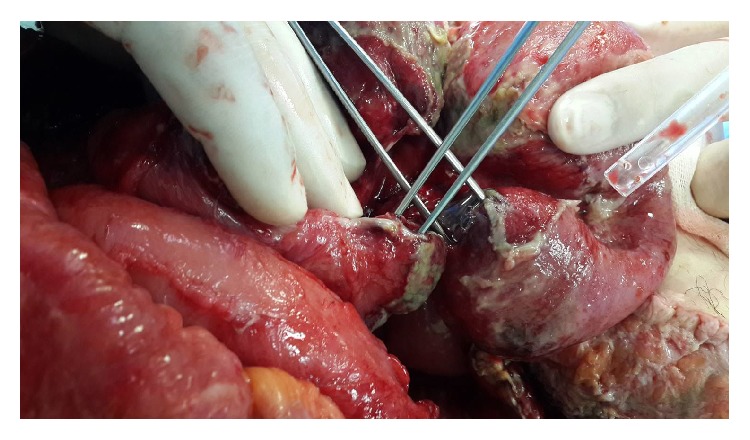
Proximal and distal part of perforation point on jejunum are seen.

**Figure 7:**
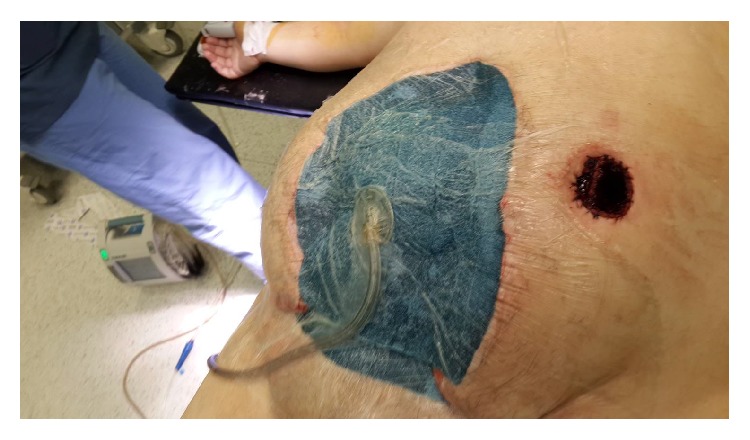
Double barrel jejunostomy and NPT application are seen.

Two days after NPT application, although source control could not be performed, vital parameters of the patient were better and dose of vasoactive support was decreased. T was 110/65 mmHg, HR was 115, RR was 26, and F was 37.1°C. The improvement of septic parameters of patient was shown in Figures 1–3 and SOFA score and expected mortality were decreased from 12 to 8 and 45% to 20%, respectively. The patient underwent operation again. Enteric influents were seen (Figure 5). After gentle dissection, a perforation point on jejunum, 100 cm distal to Treitz ligament, firmly adhesive to posterior retroperitoneal part was seen (Figure 6). EAF was controlled by opening double barrel jejunostomy very hardly. NPT was applied again (Figure 7).

**Figure 8:**
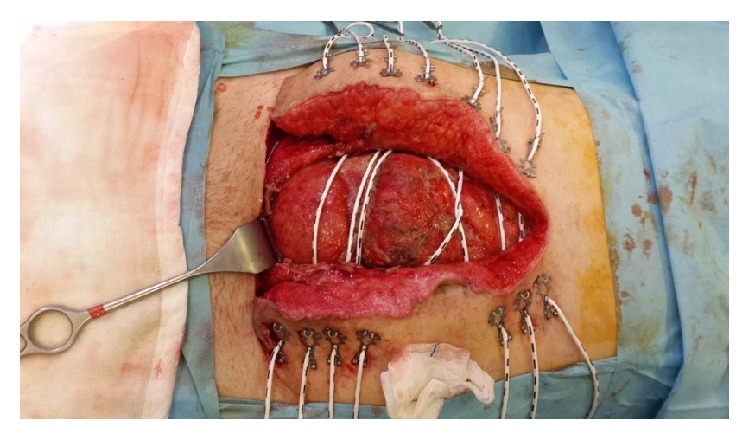
Applied ABRA is seen.

**Figure 9:**
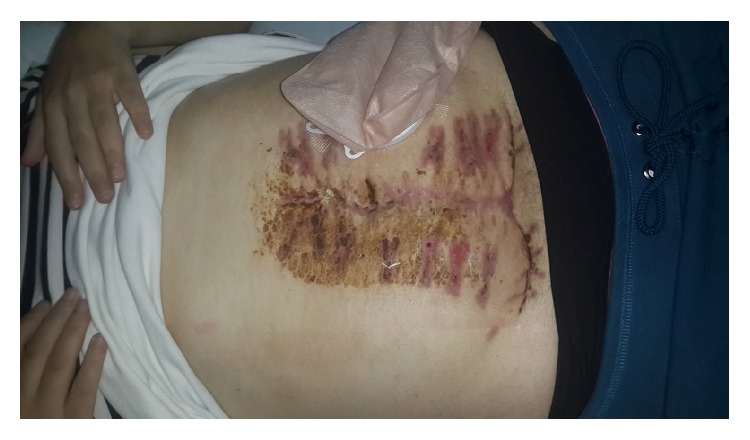
Completely closed OA is seen.

**Figure 10:**
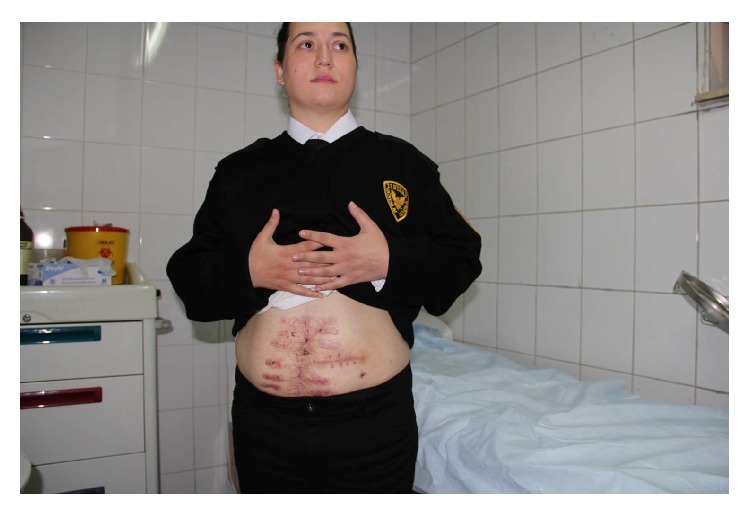
After 10 months optimum abdominal closure was achieved and there is no hernia.

SOFA score decreased to 5 at postoperative 9th day with improvement in all parameters (Figures 2–4). Following intra-abdominal source control, ABRA was added to close both the fascia and skin (Figure 8). Abdominal NPT dressing and ABRA arrangement were changed every 2–4 days. Fascia was closed approximately 15 days later with 1/0 PDS and skin was closed 3 days later. Delayed closure of OA was completed after anchors of ABRA were removed 3–6 days later (Figure 9). The patient was discharged from the hospital at postoperative 35th day. During this management operations were performed 11 times for the change of the NPT dressing system and ABRA rearrangement. Lomotil 4*∗*2 as well as 1000 cc intravenous fluid intake a day was ordered. The distal limp of ostomy was also used for enteral nutrition especially for fluid replacement. Additional to oral intake 500 cc %10 dextrose as well as 500 cc %09 NaCl isotonic solution was given daily to the distal limp. By using the distal limp of ostomy, it was understood that the passage of the distal part of the intestine was present. Two months later patient was hospitalized and operated on for closure of the jejunostomy. The distal limp was irrigated with 2000 cc NaCl isotonic solution before the surgery. Only elliptic incision around the stoma was used, and two ends of jejunum were isolated by gentle dissection. Side-to-side jejunojejunostomy anastomosis was performed. No hernia development was observed at 10-month follow-up period (Figure 10).

The technique of NPT was as follows: NPT (ABThera*™* Open Abdomen Negative Pressure Therapy System (KCI)) was used. After debridement and irrigation of OA with warm saline, a perforated silicone sheet was placed over the bowel under the fascia. Then sponge was placed over the silicone sheet. At the end suction tubing was applied. NPT was adjusted in the range of 50–100 mmHg continuously or intermittently [11].

The technique of abdominal reapproximation anchor system (ABRA, Canica, Almonte, Ontario, Canada) was as follows: ABRA was applied through the full thickness of the abdominal wall at a distance of approximately 5 cm from the medial fascial margin; a series of midline crossing elastomers are inserted. They are aligned about 3–5 cm apart cross the defect and fixed to button anchors at both sides of OA. The optimal tension was obtained by stretching the elastomers 1.5–2 their tension-free length.

## 3. Discussion

In a review of the literature, a total of 20 cases of post-C/S spontaneous cecal perforation were identified. Mortality rates from a cecal perforation range from 30% to 72% [4]. ECF can also develop as a result of the lesser sac inflammation through the leaves of the transverse mesocolon or thrombosis of adjacent mesenteric vessels and infarction [5]. Even if it is the mild form, AP is one of the factors making the management of OA patient challenging. In our case, because of severe adhesions it was impossible to touch this part of jejunum during C/S. There could be several factors for occurrence of spontaneous jejunal perforation following C/S far from operation side. These are weakening this part of jejunum due to mild acute pancreatitis, fixation of it to retroperitoneal area firmly due to previous surgery, and traction or pressure forces applied to this part of jejunum during CPR or C/S.

During emergent induction of patient with acute pancreatitis for delivery of death fetus, both these conditions may cause intracellular volume deficiency separately. Arrest on induction may develop due to insufficient preinduction resuscitation as in our case.

In severe hemodynamic instability and physiologic derangements definitive repair and definitive closure could not be obtained and restoration of intestinal continuity is postponed to other operations [12]. As far as we are concerned, NPT provided improvement in all sepsis related biomarkers and vital parameters of the patient, although surgery had to be finished without source control in the first operation. Kubiak et al. demonstrated in ischemia reperfusion of intestine and intra-abdominal sepsis model of multiple organ injury that application of peritoneal NPT model significantly reduced lung, kidney, liver, and intestinal pathology and improved pulmonary parameters by the mechanism of peritoneal cytokine removal [13]. This case is just like a clinical application of Kubiak's study expressing that the idea of using NPT is a viable modality to treat complex septic and trauma patients at risk of developing MODS without source control achievement.

Lots of different methods by combining NPT to other delayed closure methods or a novel device are used for the management of Open Abdomen with EAF [7, 8]. In Open Abdomen EAFs are controlled by converting the fistula to a stoma or ECF. Stoma, especially loop stoma, is preferred during Open Abdomen management [14]. In this case EAF was converted to loop jejunostomy. This case had short bowel syndrome with 100 cm jejunum. The short bowel syndrome was managed by giving 1000 cc liquid from distal limp of ostomy and 1000 cc IV liquid daily during this two-month period. Timing of stoma reversal is very important and difficult in this type of septic Open Abdomen patient. While delay in stoma reversal may increase stoma related complications, early reversal of stoma may increase perioperative complication ratio [14]. In this case, stoma reversal was performed 2 months after fascial closure time in order to decrease stoma related complications.

When NPT was combined with the strategies allowing reapproximation of the fascial edges, high closure rates can be achieved [7, 8, 10, 15]. Use of mesh-mediated fascial traction methods may be more suitable in noninfected OA patients, whereas ABRA can be used in the severely infected OA patients in conjunction with NPT. Pressure sores on skin may develop by transmural traction on the buttons or anchors during ABRA application [11, 16]. In our case, superficial pressure sores also developed and eventually healed well.

## 4. Conclusion

NPT application in OA patient with EAF may gain time to patient as well as to surgeon until adequate source control could be achieved. Optimal fascial closure of patient with septic OA without mesh could be done by using dynamic abdominal closure methods in conjunction with NPT.
